# Mitochondrial proteomic adaptations to daily torpor in the Djungarian hamster (*Phodopus sungorus*)

**DOI:** 10.1007/s00360-025-01625-0

**Published:** 2025-07-15

**Authors:** Anna Kovacs, Rob H. Henning, Hjalmar Permentier, Justina C. Wolters, Annika Herwig, Hjalmar R. Bouma

**Affiliations:** 1 Department of Clinical Pharmacy and Pharmacology, University Medical Center Groningen (UMCG), University of Groningen, Groningen, The Netherlands; 2 Department of Internal Medicine, University Medical Center Groningen (UMCG), University of Groningen, Groningen, The Netherlands; 3 Interfaculty Mass Spectrometry Center, Groningen Research Institute of Pharmacy, University of Groningen, Groningen, The Netherlands; 4 Department of Pediatrics, University Medical Center Groningen (UMCG), University of Groningen, Groningen, The Netherlands; 5 Institute of Comparative Molecular Endocrinology, Ulm University, D-89081 Ulm, Germany; 6 Department of Acute Care, University Medical Center Groningen (UMCG), University of Groningen, Groningen, The Netherlands

**Keywords:** Daily torpor, Hibernation, Mitochondrial proteomics, Energy metabolism, Djungarian hamster

## Abstract

Hibernation is an adaptive strategy that conserves energy in response to environmental challenges. While mitochondrial proteomic adaptations are well-documented in deep hibernators, the proteomic changes underlying daily torpor remain less clear. We investigated mitochondrial proteomic adaptations in the liver of a daily hibernator, the Djungarian hamster *(Phodopus sungorus)*, across different hibernation phases. Hamsters were maintained under long-day (summer) or short-day photoperiods (winter), to induce torpor. Livers from summer, torpor, and interbout euthermia phases were analyzed by liquid chromatography-mass spectrometry with labelled standards of mitochondrial energy metabolism proteins, resulting in accurate quantitative proteomics. Differential protein regulation was assessed using empirical Bayes models with false discovery rate correction. Increased abundance of fatty acid oxidation enzymes during hibernation indicates a seasonal metabolic shift toward lipid utilization, similar to deep hibernators. Additionally, torpor featured elevated complex II subunits and tricarboxylic acid cycle enzymes representing evolutionary adaptations specific to daily torpor, likely to cater higher energy demands necessary to maintain torpid body temperature above 15 °C in near-freezing ambient temperatures. This represents evolutionary adaptations specific to daily torpor. Increased levels of the mitochondrial uncoupling-related solute carrier family 25 member 5 (SLC25A5) may be responsible for both thermogenesis and limiting production of reactive oxygen species. Furthermore, the selective upregulation of SOD2 during torpor underscores its critical role in mitigating reactive oxygen species accumulation during metabolic transitions. In summary, daily torpor exhibits unique mitochondrial proteomic adaptations that distinguish it from deep torpor, which may be necessary to enable torpor at body temperatures well above the ambient temperature.

**Supplementary Information:**

The online version contains supplementary material available at 10.1007/s00360-025-01625-0.

## Introduction

Torpor is an adaptive response to survive periods of low food supply by reducing metabolic rate. Hibernation involves alternating cycles of torpor and interbout euthermia. Periods of torpor are marked by metabolic suppression, a drop in body temperature, and a cessation of locomotion, resulting in reduced energy consumption. These energy-saving torpor bouts are interspersed with shorter, energetically costly interbout euthermia (IBE) phases, where metabolism is restored and body temperature increases to summer levels. There are two main forms of hibernation: deep hibernation and daily hibernation, which differ in their duration and the accompanying physiological changes (Jastroch et al. 2016). Deep seasonal hibernators, such as ground squirrels, undergo prolonged, multi-day torpor bouts, alternating with brief IBE phases (Jastroch et al. 2016). During deep torpor small rodents’ body temperature drops close to ambient levels and may even fall slightly below freezing (Bouma et al. 2013). In contrast, daily heterotherms, such as the Djungarian hamster *(Phodopus sungorus)* and certain bats, experience short episodes of torpor within a 24-hour cycle, typically lasting a few hours with body temperatures typically between 15–25ºC (Bouma et al. 2013; Jastroch et al. 2016). Daily torpor can be used spontaneously in a seasonal context or as a response to immediate environmental challenges, such as food scarcity, and involves less extreme physiological changes compared to deep hibernation (Jastroch et al. 2016). Interestingly, some species, such as the edible dormouse (*Glis glis*), exhibit both forms of hibernation: daily and deep torpor (van Breukelen and Martin 2015). Both forms of hibernation rely on mitochondrial adaptations to reduce energy expenditure while maintaining cellular integrity.

Mitochondrial oxidative phosphorylation (OXPHOS) serves as the primary source of cellular ATP production and plays a crucial role in maintaining body temperature via proton leak. In seasonal hibernators, particularly during deep torpor, extensive research has highlighted key mitochondrial adaptations. One key adaptation is a reduction in OXPHOS during torpor (Armstrong and Staples 2010; Ballinger et al. 2016), which lowers the production of reactive oxygen species (ROS) (Zhao et al. 2019). Moreover, during deep torpor, mitochondria undergo architectural changes—such as increased lipid droplet interactions and a shift from a round to a crescent shape—that favor fatty acid oxidation over carbohydrate metabolism (Fedorov et al. 2009). Proteomic studies in 13-lined ground squirrels *(Ictidomys tridecemlineatus)* and black bears *(Ursus americanus)* reveal upregulation of proteins involved in lipid translocation, β-oxidation, and fatty acid metabolism during hibernation (both torpor and interbout euthermia), reflecting a seasonal shift from carbohydrate metabolism in summer to fatty acid oxidation during hibernation (Ballinger et al. 2016; Fedorov et al. 2009; Hindle et al. 2011; Jastroch et al. 2016).

In contrast to adaptations associated with deep torpor, much less is known about the mitochondrial adaptations of daily torpor, particularly at the proteomic level, although profound changes in mitochondrial function during daily torpor have been documented. Daily torpor in Djungarian hamsters’ (*Phodopus sungorus)* was found to be associated with a 30% reduction in OXPHOS capacity, along with increased mitochondrial proton leak in the liver, which presumably helps mitigate excessive mitochondrial membrane potential (ΔΨ) and reduces ROS production (Staples and Brown 2008). These mitochondrial changes resemble those linked to lower oxidative stress and increased longevity in other species, highlighting the critical role of mitochondrial regulation during torpor. Additionally, metabolic substrate utilization shifts during torpor, with glucose serving as the primary fuel during its initial phase, followed by a transition to lipid oxidation later in torpor (Heldmaier et al. 1999). Most studies on daily torpor have predominantly examined mitochondrial metabolic function (Staples and Brown 2008), while molecular changes in mitochondria remain unclear, leaving significant gaps in our understanding of how daily heterotherms regulate mitochondrial function at the molecular level. To address this gap, we performed a targeted mitochondrial proteomics analysis on liver samples from the Djungarian hamster to uncover the proteomic changes that enable daily torpor. Proteomics were done on the liver as it plays a central role in systemic metabolic regulation, including glucose and lipid homeostasis.

## Methods

### Animals

Male and female Djungarian hamsters were raised at the Rowett Institute for Nutrition and Health, University of Aberdeen (UK). The study was conducted under the Animals (Scientific Procedures) Act of 1986. Before the experiments, the animals were maintained at an ambient temperature of 21 ± 1 °C under a long-day photoperiod (16 h light: 8 h dark). To induce short day acclimatization (winter-adaptation) and elicit daily torpor, photoperiod was shifted to a short-day cycle (8 h light: 16 h dark) for approximately 14 weeks, with the temperature maintained at 21 °C. The presence of daily torpor was assessed by observing characteristic torpid behaviour, including inactivity and a distinctive torpor posture, during the middle of the light phase, which is the typical torpor period for this species. To clarify terminology, we use “torpor” to refer to the torpid hypometabolic state itself, and “hibernation” to describe the overall process comprising repeated cycles of torpor and interbout euthermia (IBE). Djungarian hamsters were euthanized either during long-day (LD) conditions, torpor (T), or interbout euthermia (IBE). For torpor (T) animals, euthanasia was performed during the torpor nadir at Zeitgeber time (ZT) 4–5, corresponding to 4–5 h after torpor entry, when torpor is typically deepest and most stable. IBE animals were euthanized at ZT 12–13, approximately 4–5 h after arousal from torpor, to ensure they were fully normometabolic. These time points were selected based on the strong circadian control of torpor in Djungarian hamsters, where torpor entry generally occurs with lights on and arousal just prior to lights off. Animals were euthanized via CO₂ exposure (taking approximately 4 min for control and IBE groups; approximately 8 min for the torpor group), followed by decapitation. After decapitation, rectal body temperature was measured. Liver sections were then sampled, snap frozen on dry ice and stored at -80 °C until further analysis.

### Targeted proteomics

The targeted proteomics carried out focused on mitochondrial energy metabolism pathways—TCA cycle, fatty acid β-oxidation, oxidative phosphorylation, and ROS detoxification—providing a tool to uncover mitochondrial regulatory adaptations. The targeted proteomics precisely quantified key regulatory proteins to enable detailed analysis of mitochondrial energy pathways while accurately detecting low-abundance proteins typically obscured in standard proteomics. The original method (Wolters et al. 2016) was designed to target the mitochondrial proteins for human, mouse and rat proteins. For this study, the selected peptides were compared to the Syrian hamster database (*Mesocricetus auratus*, UniProt, 33875 entries) to check the overlap of these sequences with the hamster proteome. This resulted in the quantification of 40 proteins (Supplementary material: Appendix 1). Quantification of these proteins was performed using the exact same quantitative targeted LC-MS based proteomics workflow after in-gel digestion applying isotopically-labeled standards for the quantification as described by Wolters et al. 2016. In short, liver extracts were prepared from snap-frozen sections, by grinding into a powder, and homogenizing in a sucrose-Tris buffer containing protease inhibitors at 4 °C. For liver samples, 25 µg of total protein content was mixed with 31.25 ng isotopically labelled protein standards (QconCATs containing concatenated peptides for mitochondrial target proteins, Polyquant Germany) in LDS loading buffer, briefly run into a precast Bis-Tris gel, and stained with Coomassie to isolate the total protein fraction from interfering non-protein components. The gelbands containing all proteins were excised, gel pieces were sequentially washed with acetonitrile and ammonium bicarbonate, reduced with dithiothreitol, alkylated with iodoacetamide, and digested overnight with trypsin, as proteolytic cleavage into peptides is a prerequisite for LC-MS-based proteomic quantification. Peptides were extracted using acetonitrile and formic acid, purified with a C18 cartridge, and the eluted fractions were dried and resuspended in formic acid for analysis. The proteins were quantified in a single measure by targeted liquid chromatography-mass spectrometry (LC-MS) assays in the selected reaction monitoring (SRM) mode. Internal standards were added in equal amounts to all samples to ensure accurate quantification and monitor technical consistency. Their stable signals across all samples confirmed reliable sample preparation and LC-MS performance, supporting that observed variation reflects true biological differences rather than technical artifacts. The accurate concentration of the endogenous peptides was calculated by comparing the sum of the peak areas between the endogenous and concatemer peptides.

### Statistical analysis

Proteomics data were analyzed using RStudio (version 4.3.1). We normalized to mitochondrial protein abundances to citrate synthase (CS) protein levels, a widely used marker of mitochondrial content (Lanza and Nair 2009; Short et al. 2005; Vigelsø et al. 2014). This approach isolates functional adaptations within the mitochondrial proteome, independent of potential fluctuations in tissue mitochondrial content. The Limma package was used for analysis. Protein levels were fitted to a linear model using a design matrix based on hibernation phases. Biologically meaningful contrasts (Long-day (LD) vs. Torpor (T), T vs. Interbout euthermia (IBE), IBE vs. LD) were defined to compare protein levels across different phases. Differential levels were assessed using an empirical Bayes approach (Kammers et al. 2015), and results were adjusted for multiple comparisons using the false discovery rate (FDR). Significance was defined as FDR < 0.05, regardless of protein abundance fold-change.

### Data availability

The targeted mass spectrometry proteomics data have been deposited on the PASSEL server with the data identifier PASS05906. https://db.systemsbiology.net/sbeams/cgi/PeptideAtlas/PASS_View.

## Results

The study included a total of 19 animals, with 63% being male (Table 1). The median age of the animals across the different experimental groups ranged from 21.9 weeks in the IBE group to 29.1 weeks in LD group. Torpor was confirmed by a significantly lower body temperature of 24 °C as compared to ~ 35 °C in LD and IBE animals.

**Table 1:** Population characteristics of the Djungarian hamster (*N* = 19)

	Summer-adapted	Winter-adapted	
	Long-day (LD) *N* = 5	Torpor (T) *N* = 9	Interbout euthermia (IBE) *N* = 5
Sex male (N (%))	4 (80.0)	5 (55.5)	3 (60.0)
Age (weeks, median (IQR))	29.1 (26.6–30.3)*	22.1 (18.9–22.4)	21.9 (17.6–22.1)
Body temperature (℃, mean (± SD))	35.2 (0.2)	24.0 (0.7)*	35.6 (0.7)

* Value significantly different (*p* < 0.05) compared to both other groups

**Fig. 1:**
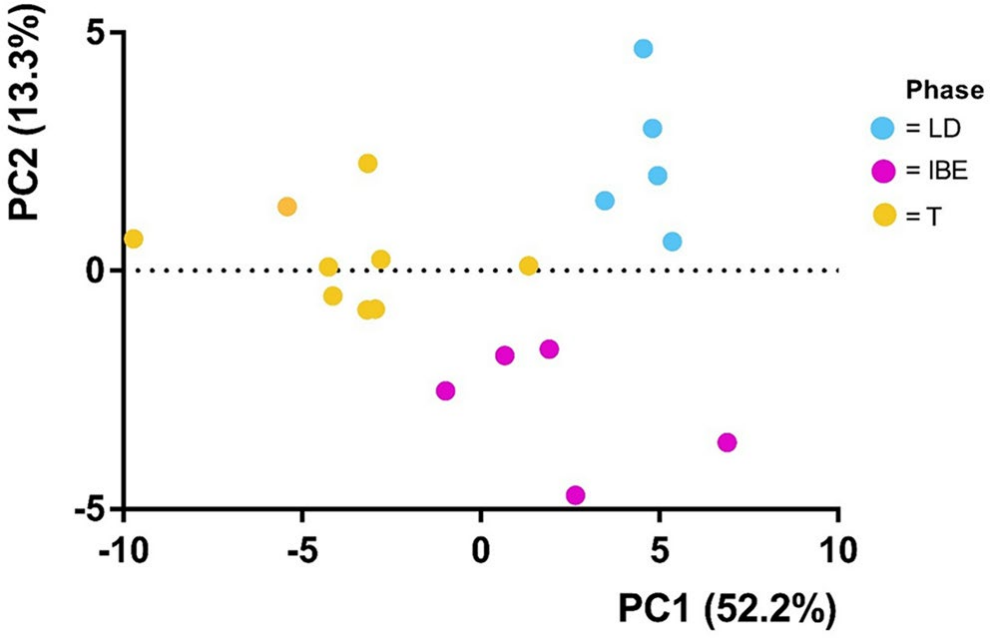
Principal component analysis (PCA) of mitochondrial protein abundance across different seasons and metabolic states. PCA1, which explains 52.2% of variance in the data, is plotted against PCA2, which explains 13.3% of the variance. The animals (points) are clustered based on their protein abundance profiles. Colors indicate the phase of hibernation: LD = long-day, IBE = Interbout euthermia, T = torpor

Targeted proteomics of 40 proteins specific to mitochondrial metabolism was performed on snap-frozen liver tissue (Supplementary material: Appendix 1). Principal component analysis (PCA) revealed distinct clustering patterns based on mitochondrial protein abundance across different phases (Fig. 1). PC1 accounted for 52.2% of the total variance and primarily separated torpor (T) from both IBE and LD, suggesting that mitochondrial protein regulation in torpor differs greatly from the other two phases. PC2 explained 13.3% of the variance and separated IBE from LD. Together, these two principal components captured 65.5% of the variance, demonstrating clear phase-dependent differences in mitochondrial protein abundance.

**Fig. 2:**
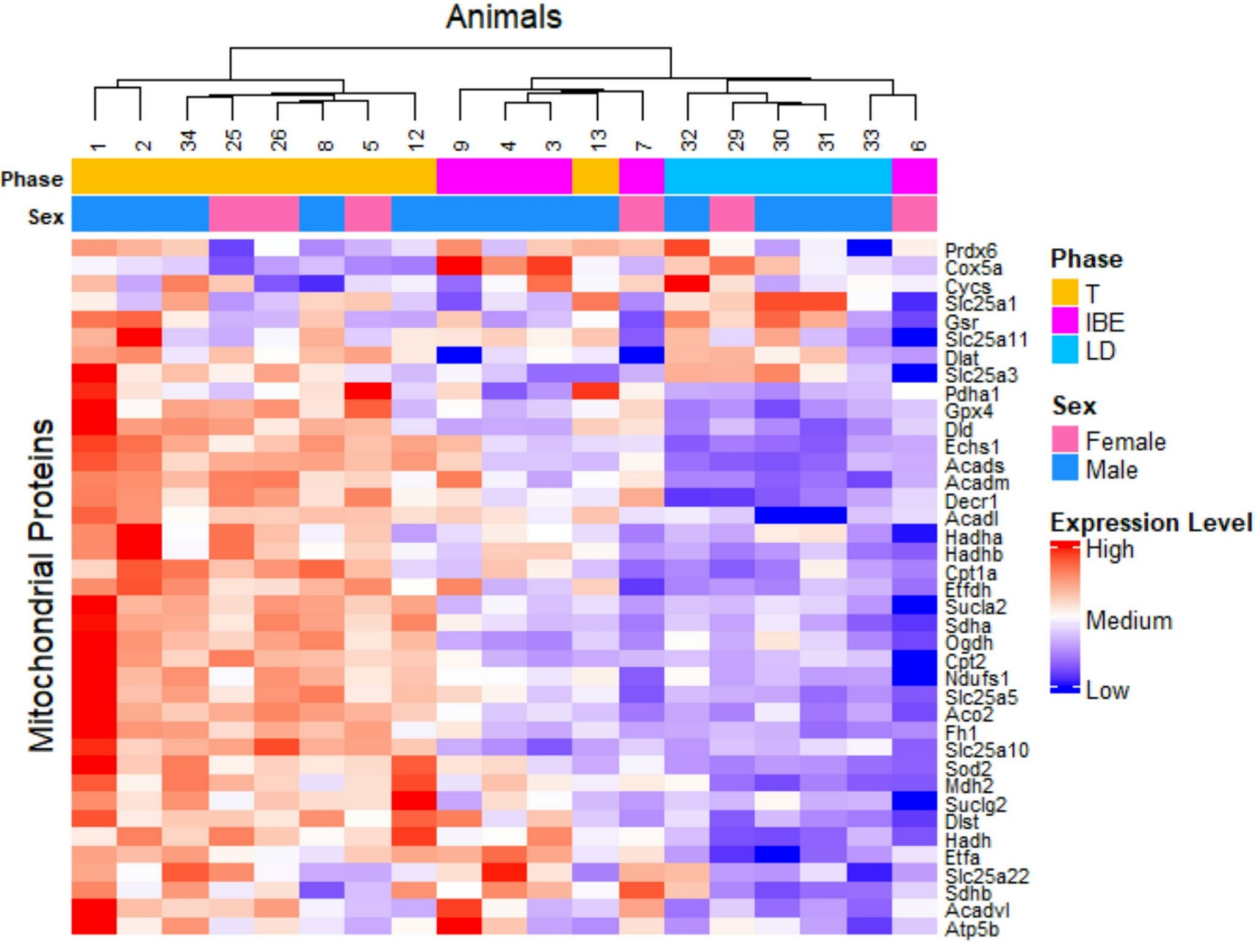
Heatmap of the mitochondrial protein abundances across different seasons and metabolic states. The animals (columns) are clustered by their protein abundance profile (rows). Colors indicate protein abundance, ranging from high (red) to low (blue). Animal sex and phase of hibernation are annotated at the top of the heatmap

Normalized mitochondrial protein abundance is depicted in a heatmap (Fig. 2), which shows clustering of animals within hibernation phases. In particular, most mitochondrial proteins show increased abundance in torpor compared to IBE and LD. Based on the heatmap, the clustering of the LD animals seems to be the most coherent group in terms of their mitochondrial protein profile.

**Fig. 3:**
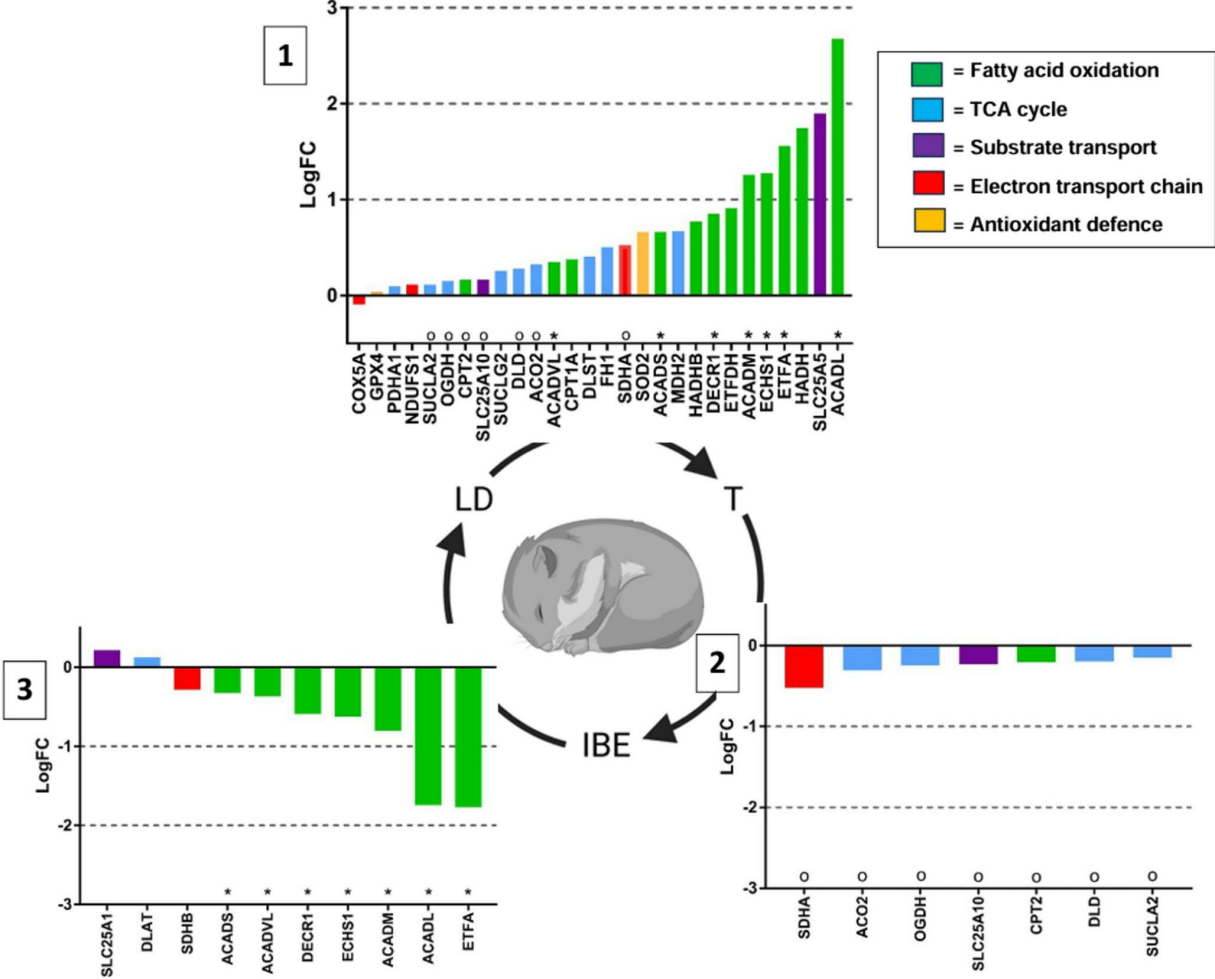
Changes in mitochondrial protein abundance across different seasons and metabolic states in the Djungarian hamster: torpor (T), interbout euthermia (IBE), and long-day (LD). Only proteins that exhibit significant differences between phases are displayed for each contrast (FDR < 0.05). Bar charts depict the log-fold changes (LogFC) in protein abundance, with bars color-coded according to the biological pathway associated with each protein. Symbol ‘o’ is inserted above the x-axis for proteins that are oppositely regulated between panel 1 and 2. Symbol ‘*’ is inserted above the x-axis for proteins that are oppositely regulated between panel 1 and 3

The abundance of 28 proteins differed between torpor and LD, 7 between torpor and IBE, and 10 between IBE and LD (Fig. 3). The observed changes in protein abundance can be categorized into two main patterns: The first pattern represents hibernation changes, which are present during both torpor and IBE compared to LD animals. Notably, many proteins involved in fatty acid oxidation, including electron transfer flavoprotein subunit alpha (ETFA), very long-chain acyl-CoA dehydrogenase (ACADVL), long-chain acyl-CoA dehydrogenase (ACADL), medium-chain acyl-CoA dehydrogenase (ACADM), short-chain acyl-CoA dehydrogenase (ACADS), enoyl-CoA hydratase 1 (ECHS1), and 2,4-dienoyl-CoA reductase 1 (DECR1) showed higher abundance during hibernation compared to summer (Fig. 3).

Secondly, there are phase-specific changes, which occur exclusively during either IBE or torpor. Torpor-specific changes include an increased abundance of multiple TCA cycle enzymes, such as pyruvate dehydrogenase E1 alpha 1 subunit (PDHA1), succinyl-CoA ligase ADP-forming beta subunit (SUCLA2), oxoglutarate dehydrogenase (OGDH), succinyl-CoA ligase GDP-forming alpha subunit (SUCLG2), dihydrolipoamide dehydrogenase (DLD), aconitase 2 (ACO2), dihydrolipoamide S-succinyltransferase (DLST), fumarate hydratase (FH1), and malate dehydrogenase 2 (MDH2) (Fig. 3). Additionally, the substrate transporter solute carrier family 25 member 5 (SLC25A5), responsible for ADP import into and ATP export out from mitochondria, had a 1.9 times higher abundance in torpor than in LD animals. Furthermore, torpor features increased levels of SOD2 antioxidant enzyme. Among electron transport chain complexes, complex II (succinate dehydrogenase flavoprotein subunit A [SDHA], succinate dehydrogenase iron-sulfur subunit B [SDHB]) undergoes the strongest regulation, with its abundance increasing during torpor, decreasing during the transition from torpor to IBE, and further declining when transitioning from IBE to LD.

In addition, during the transition from torpor to IBE, a pronounced decrease in protein abundance was observed, particularly in TCA cycle enzymes (ACO2, OGDH, DLD, SUCLA2), which had increased abundance during torpor (Fig. 3). Additionally, the fatty acid oxidation enzyme carnitine O-palmitoyl transferase 2 (CPT2) and the substrate transporter solute carrier family 25 member 10 (SLC25A10), which facilitates the exchange of malate and succinate for phosphate, also showed reduced protein levels. As animals transition from IBE to LD, most of the fatty acid oxidation enzymes that were elevated during hibernation show a decline in abundance (Fig. 3).

## Discussion

**Fig. 4:**
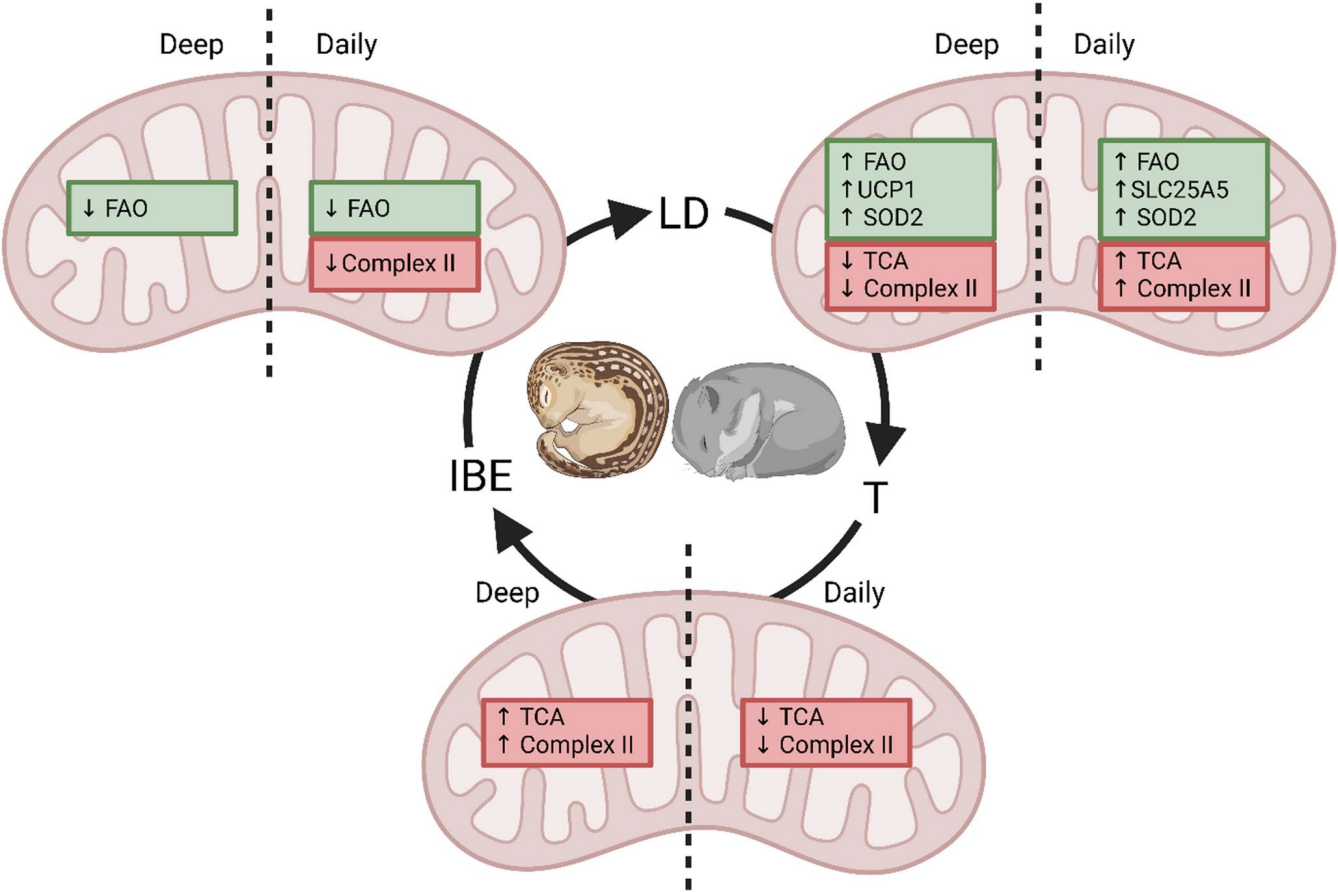
Schematic representation of the mitochondrial functional regulation in daily heterotherms (Djungarian hamsters, this study) versus deep hibernators (13-lined ground squirrels, literature*) across metabolic states: long-day control (LD), torpor (T), and interbout euthermia (IBE). Green boxes denote shared regulatory patterns, while red boxes indicate species-specific differences. Abbreviations: FAO: fatty acid oxidation; TCA: tricarboxylic acid cycle. *Literature used to create this figure: (Ballinger et al. 2016; Green and Storey 2020; Hampton et al. 2013; Laursen et al. 2015; Mathers et al. 2017; Mathers and Staples 2019; Vucetic et al. 2013; Wijenayake et al. 2017; Yan et al. 2006, 2008)

This study utilized a targeted mitochondrial proteomics approach to measure levels of key mitochondrial proteins regulated during daily torpor in the Djungarian hamster. Targeted proteomics was chosen for its higher sensitivity, as the use of concatemers allows for the detection of low-abundance proteins that might otherwise go unnoticed. Additionally, unlike shotgun proteomics, targeted approaches provide accurate quantification of protein levels in mitochondrial protein regulation between different states, decreasing the variation in the measurements as well as a wider dynamic range for quantification. The results reveal hibernation and phase-dependent regulation of mitochondrial proteins, highlighting both conserved and divergent patterns relative to deep hibernators, as illustrated in Fig. 4 and discussed in the following paragraphs.

### Increased fatty acid beta-oxidation enzymes during hibernation

Fatty acid oxidation appears to play a crucial role in energy production during hibernation compared to the summer phase (Hindle et al. 2011). Levels of fatty acid oxidation enzymes, including ACADS, ACADM, ACADL, ACADVL, DECR1, ECHS1, and ETFA, were higher during both torpor and IBE compared to LD. Acyl-coenzyme A dehydrogenases (ACADS, ACADM, ACADL, and ACADVL) catalyze the initial step of mitochondrial fatty acid oxidation (Bartlett and Eaton 2004), while DECR1 is the rate-limiting enzyme in polyunsaturated fatty acid oxidation (Nassar et al. 2020). ECHS1 plays a dual role: on the one hand, facilitating the hydration of medium- and short-chain fatty acids (C4-C6) during β-oxidation, and other hand contributing to the degradation of branched-chain amino acids (Napoli et al. 2020). After fatty acids are oxidized, ETF (measured here by the subunit alpha ETFA) shuttles electrons acquired from fatty acid oxidation into the electron transport chain at complex III (Henriques et al. 2021). The increased abundance of fatty acid oxidation enzymes observed in the Djungarian hamster during hibernation mirrors similar findings in the brown adipose tissue (BAT) of deep hibernators like the 13-lined and Arctic ground squirrels *(Urocitellus parryii)* (Ballinger et al. 2016; Hampton et al. 2013; Yan et al. 2006). Although daily heterotherms feed more frequently during interbout euthermia than deep hibernators, they consistently rely on fatty acid oxidation to maintain energy homeostasis during torpor when food intake is reduced. While the increased abundance of fatty acid oxidation enzymes likely reflects a greater reliance on lipid metabolism, an alternative possibility is that it compensates for temperature-dependent reductions in enzymatic activity. If fatty acid oxidation enzymes are more temperature-sensitive (Q10) than glycolytic enzymes, higher enzyme levels may be necessary to sustain metabolic flux at lower body temperatures. However, this remains speculative, as data on the Q10 values of these enzymes in rodents are currently lacking.

### Increased TCA cycle enzyme levels in torpor

In Djungarian hamsters, nine of ten quantified mitochondrial enzymes involved in the TCA cycle—namely MDH2, FH1, DLST, PDHA1, SUCLA1, OGDH, SUCLG2, DLD, and ACO2—exhibit increased abundance during torpor. The likely physiological driver is the thermoregulatory constraint of daily torpor: unlike deep hibernators, which tolerate core body temperatures near 0 °C, daily heterotherms maintain body temperatures above ~ 15 °C during torpor despite subzero ambient conditions (Bouma et al. 2013; Jastroch et al. 2016). This imposes a continuous energetic demand, requiring sustained mitochondrial ATP production to support gluconeogenesis, biosynthesis, and non-shivering thermogenesis (Bertile et al. 2021). Accordingly, increased TCA enzyme abundance may ensure adequate fuel for the electron transport chain under these metabolically active but low-temperature conditions. In contrast, deep hibernators such as the 13-lined ground squirrel display a more variable TCA enzyme profile. While enzymes such as OGDH, DLST, DLD, PDHA1, and MDH2 increase in abundance during torpor (Ballinger et al. 2016; Green and Storey 2020; Yan et al. 2008), others-like SUCLG2 and ACO2-remain stable or decline (Ballinger et al. 2016). Recent findings indicate that deep hibernators employ a different regulatory strategy: instead of modulating TCA cycle activity primarily through changes in protein abundance, they rely on reversible post-translational modifications (PTMs) (Green and Storey 2020; Wijenayake et al. 2017). This approach allows for rapid and flexible metabolic responses (Zhong et al. 2023), which is essential as these animals markedly lower TCA cycle flux (Mathers and Staples 2019) and core body temperature during torpor, then swiftly reactivate TCA cycle enzymes to support rewarming to euthermic levels during arousal (Mathers and Staples 2019). Beyond energy production, TCA cycle enzymes also serve key redox-regulatory roles. MDH2, for instance, stabilizes glutathione peroxidase 4 (GPX4), a central regulator of lipid peroxide detoxification and ferroptosis suppression (Yu et al. 2024). OGDH, while capable of generating reactive oxygen species (ROS), acts as a redox-sensitive enzyme whose activity modulates in response to oxidative cues (Chang et al. 2022). These dual roles underscore the TCA cycle’s centrality not only in maintaining bioenergetic output but also in preserving redox homeostasis under fluctuating metabolic states.

### Increased SLC25A5 levels in torpor

SLC25A5, traditionally recognized as an ADP/ATP translocase facilitating the exchange of cytosolic ADP for mitochondrial ATP across the inner mitochondrial membrane, has also been implicated in proton leak activity, thereby contributing to mitochondrial uncoupling and thermogenesis (Bround et al. 2020). In Djungarian hamsters SLC25A5 levels are elevated during torpor. This hypothesis is corroborated by physiological data from Djungarian hamsters demonstrating increased hepatic proton leak during torpor, which subsides upon transition to IBE (Brown et al. 2007). This is a crucial adaptation for daily heterotherms, which must maintain body temperatures above 15 °C during torpor, requiring more endogenous heat production than deep hibernators (Diedrich et al. 2023). The thermogenic role of SLC25A5 is further supported by studies in the deep-hibernating 13-lined ground squirrel, which show upregulation of mitochondrial uncoupling protein 1 (UCP1) in brown adipose tissue and neurons during torpor, facilitating tissue-specific heat production (Ballinger et al. 2016; Laursen et al. 2015). Although UCP1 is not expressed in the liver, SLC25A5—sharing notable homology with UCP1 (Skulachev 1999)—may serve an analogous function in hepatic tissue (Zhang et al. 2025). Beyond its thermogenic capacity, SLC25A5-mediated proton leak also contributes to the reduction of mitochondrial membrane potential (ΔΨm), a well-established mechanism for limiting mitochondrial reactive oxygen species (ROS) production and mitigating oxidative stress (Pohl et al. 2025). Additionally, through its role as an ADP/ATP exchanger, SLC25A5 supports energy homeostasis by ensuring ATP availability for essential cellular functions, including damage repair during torpor (Tolouee et al. 2022). Together, the torpor-associated increase in SLC25A5 expression highlights a conserved mitochondrial adaptation that integrates enhanced ADP/ATP with increased proton leak to coordinate thermogenesis and redox balance.

### Complex II regulation throughout hibernation

In Djungarian hamsters there is an increased abundance of Complex II subunits SDHA and SDHB during torpor, followed by a progressive decline during IBE and control phases. Given that Complex II constitutes the primary entry point for TCA cycle-derived electrons into the respiratory chain (Esteban-Amo et al. 2024), its higher abundance is consistent with the observed enhancement of TCA cycle enzyme levels during torpor. This coordinated increase in both complex II and TCA cycle enzyme abundances likely facilitates sustained mitochondrial ATP production, thereby supporting energy-intensive processes such as hepatic gluconeogenesis (Bertile et al. 2021). The resulting glucose supply is critical for fueling thermogenic tissues like brown adipose tissue and skeletal muscle, which generate the heat necessary to maintain core body temperatures above ~ 15 °C in daily heterotherms exposed to subzero ambient conditions (Bouma et al. 2013; Jastroch et al. 2016). However, despite this increase in Complex II abundance, a previous study have reported reduced Complex II activity during daily torpor in both mice and Djungarian hamsters (Brown and Staples 2011). The observed discrepancy between increased protein abundance in Djungarian hamsters found in this study and reduced activity seen previously may reflect differences in readouts (i.e., levels versus activity). Similarly, deep hibernators, like the 13-lined ground squirrel, suppress Complex II activity by 30% during torpor through post-translational modifications such as phosphorylation (Mathers et al. 2017; Mathers and Staples 2019) and allosteric regulation by oxaloacetate (Armstrong and Staples 2010). This allows for metabolic suppression while preserving the capacity for rapid enzymatic reactivation during IBE (Zhong et al. 2023), when core body temperature rises sharply from near 0 °C to ~ 30 °C. Thus, although the modes of regulation differ—gene-level control in daily torpor and enzyme-level control in deep hibernation—both strategies serve the same purpose: adjusting mitochondrial activity to meet the unique thermal and metabolic needs of each species.

### Higher SOD2 levels in torpor

Among all antioxidant enzymes analyzed, only SOD2 was upregulated during torpor, underscoring its specific role in mitigating ROS accumulation and maintaining redox homeostasis during metabolic transitions in hibernating mammals. Despite steady H₂O₂ levels reported during hibernation (Wei et al. 2018)—indicating efficient ROS removal mechanisms that may protect tissues during IBE —oxidative damage does occur. For instance, lipid peroxidation, measured by malondialdehyde (MDA) in isolated mitochondria, more than doubles during hibernation (Gerson et al. 2008). Consistent with this, increased SOD2 activity has been observed in multiple hibernating species. In European ground squirrels (*Spermophilus citellus*), SOD2 activity in BAT increases during hibernation (Vucetic et al. 2013). Similarly, elevated SOD2 levels were detected in the liver of Daurian squirrels (*Spermophilus dauricus)* during early torpor and IBE (Wei et al. 2018). These findings collectively underscore the importance of SOD2 in mitigating oxidative stress during deep torpor as well as daily torpor, protecting tissues from damage.

### Analytical approach

It is important to note that the significance of differentially abundant proteins in this study was determined using a false discovery rate (FDR) cutoff < 0.05. In some proteomics studies, an additional threshold is applied, requiring a minimum fold-change of 50% abundance (|LogFC| ≥ 0.59) to define biologically meaningful changes. While such stringent approaches are relevant for limiting overfitting in large datasets, we consider such a stringent approach not applicable to the current analysis, since we measured a relatively small set of predefined proteins (*n* = 40). While the targeted proteomics approach used in this study offers high sensitivity and specificity for quantifying selected mitochondrial proteins, it inherently narrows the scope of analysis. By focusing on a predefined set of proteins, other potentially important proteins or pathways involved in metabolic adaptation during hibernation may be overlooked. Future studies may benefit from complementing this targeted approach with global proteomic or transcriptomic analyses to capture an extended view of the molecular changes occurring during hibernation. Then, the signaling pathways leading to these proteomic changes may also be studied in more detail.

A limitation is that we normalized mitochondrial proteomic data to citrate synthase (CS) protein levels, a widely used approach to account for differences in mitochondrial number between samples and isolate true mitochondrial proteomic adaptations (Lanza and Nair 2009; Short et al. 2005; Vigelsø et al. 2014). Moreover, evidence from the Richardson’s ground squirrels demonstrates that CS levels remain stable across torpor-IBE cycles (Green and Storey 2020). However, we cannot completely rule out the possibility of CS regulation in hibernating Djungarian hamsters.

In addition, while proteomics cannot directly differentiate between protein synthesis and degradation, evidence from both daily and deep hibernators indicates that torpor involves a coordinated suppression of protein turnover. In Djungarian hamsters, transcriptional initiation has been shown to decrease by 40% alongside reduced protein synthesis (Berriel Diaz et al. 2004), while studies in golden-mantled ground squirrels show that low temperatures inhibit proteasome activity and protein degradation (Velickovska et al. 2005). Together, these adaptations maintain proteostasis and conserve energy during torpor.

Although our study was not designed to assess sex-specific effects (12 males, 7 females), we acknowledge that sex hormones may modulate metabolic responses during torpor. Prior work in hibernating bears demonstrated sex-dependent regulation of androstenedione and testosterone, with opposing patterns in males and females (Frøbert et al., 2022). Similarly, in common hamsters (Cricetus cricetus), females exhibit shorter hibernation durations than males (Siutz et al., 2016). Further research is warranted to explore potential sex-based regulation of mitochondrial metabolism during torpor and interbout euthermia.

In summary, this study provides new insights into the origins of differential mitochondrial adaptations in daily versus deep hibernators, demonstrating that the requirement to maintain a higher core body temperature during daily torpor is associated with distinct mitochondrial adaptations compared to deep hibernation. Our findings show that mitochondrial suppression is associated with diverse mechanisms that vary across species and hibernation types, underscoring the need for interspecies comparisons before assuming universal strategies of metabolic adaptation. Expanding mitochondrial proteomic analyses to a broader range of hibernating species will be important for elucidating both conserved and species-specific adaptations, ultimately advancing our understanding of the evolutionary and physiological diversity underlying mammalian hypometabolism and informing future biomedical research on metabolic flexibility and resilience.

## Conclusion

We investigated mitochondrial proteomic adaptations in the liver of the Djungarian hamster *(Phodopus sungorus)* during daily torpor. Increased fatty acid oxidation enzyme levels during hibernation indicate a seasonal metabolic shift toward lipid utilization. Additionally, elevated levels of TCA cycle enzymes and complex II in daily torpor may be necessary to meet the higher energetic demands of maintaining a body temperature above 15 °C in a near freezing environment—an evolutionary adaptation distinguishing daily torpor from deep hibernation. Elevated SLC25A5 levels during daily torpor may contribute to thermogenesis or help minimize ROS production, and it is thought to have functions similar to UCP1 in deep torpor. Furthermore, the exclusive upregulation of SOD2 during torpor highlights its pivotal role in antioxidant defense, specifically in mitigating ROS accumulation and preserving cellular stability during metabolic transitions. In summary, daily torpor exhibits unique mitochondrial proteomic adaptations that distinguish it from deep torpor.

## Electronic supplementary material

Below is the link to the electronic supplementary material.

Supplementary Material 1
