# Supplementary material for: Mitochondrial proteomic adaptations to daily torpor in the Djungarian hamster (Phodopus sungorus)
Source: J Comp Physiol B. 2025 Jul 15;195(4):481–91. doi: 10.1007/s00360-025-01625-0 (PMC12367877; doi:10.1007/s00360-025-01625-0)
Supplement: Supplementary file 1 — Supplementary Material 1 [file 360_2025_1625_MOESM1_ESM.docx]

| **Protein name abbreviation** | **Protein full name** | **Pathway** |
| --- | --- | --- |
| Gpx4 | Glutathione peroxidase 4 | Antioxidant |
| Gsr | Glutathione reductase | Antioxidant |
| Prdx6 | Peroxiredoxin 6 | Antioxidant |
| Sod2 | Superoxide dismutase [Mn], mitochondrial | Antioxidant |
| Ndufs1 | NADH dehydrogenase (complex I) iron-sulfur protein 1 | Electron transport chain |
| Sdha | Succinate dehydrogenase (complex II) flavoprotein subunit | Electron transport chain |
| Sdhb | Succinate dehydrogenase (complex II) iron-sulfur subunit | Electron transport chain |
| Cox5a | Cytochrome c oxidase (complex IV) subunit 5A | Electron transport chain |
| Atp5b | ATP synthase (complex V) subunit beta | Electron transport chain |
| Cycs | Cytochrome c, somatic | Electron transport chain |
| Acadl | Long-chain specific acyl-CoA dehydrogenase | Fatty acid oxidation |
| Acadm | Medium-chain specific acyl-CoA dehydrogenase | Fatty acid oxidation |
| Acads | Short-chain specific acyl-CoA dehydrogenase | Fatty acid oxidation |
| Acadvl | Very-long-chain specific acyl-CoA dehydrogenase | Fatty acid oxidation |
| Cpt1a | Carnitine palmitoyltransferase 1A | Fatty acid oxidation |
| Cpt2 | Carnitine palmitoyltransferase 2 | Fatty acid oxidation |
| Decr1 | 2,4-dienoyl-CoA reductase 1 | Fatty acid oxidation |
| Echs1 | Enoyl-CoA hydratase, mitochondrial | Fatty acid oxidation |
| Hadh | Hydroxyacyl-CoA dehydrogenase | Fatty acid oxidation |
| Hadha | Hydroxyacyl-CoA dehydrogenase trifunctional multienzyme complex subunit alpha | Fatty acid oxidation |
| Hadhb | Hydroxyacyl-CoA dehydrogenase trifunctional multienzyme complex subunit alpha | Fatty acid oxidation |
| Etfa | Electron transfer flavoprotein subunit alpha | Fatty acid oxidation |
| Etfdh | Electron transfer flavoprotein-ubiquinone oxidoreductase | Fatty acid oxidation |
| Slc25a10 | Mitochondrial dicarboxylate carrier | Substrate transport |
| Slc25a11 | Mitochondrial 2-oxoglutarate/malate carrier | Substrate transport |
| Slc25a22 | Mitochondrial glutamate carrier | Substrate transport |
| Slc25a3 | Phosphate carrier protein | Substrate transport |
| Slc25a5 | ADP/ATP translocase 2 | Substrate transport |
| Aco2 | Aconitase 2 | TCA cycle |
| Cs | Citrate synthase | TCA cycle |
| Dlst | Dihydrolipoamide S-succinyltransferase | TCA cycle |
| Fh1 | Fumarate hydratase, mitochondrial | TCA cycle |
| Mdh2 | Malate dehydrogenase, mitochondrial | TCA cycle |
| Ogdh | Oxoglutarate dehydrogenase (lipoamide) | TCA cycle |
| Sucla2 | Succinyl-CoA ligase [ADP-forming] subunit beta | TCA cycle |
| Dlat | Dihydrolipoyllysine-residue acetyltransferase component of pyruvate dehydrogenase complex | TCA cycle |
| Dld | Dihydrolipoamide dehydrogenase | TCA cycle |
| Pdha1 | Pyruvate dehydrogenase E1 component subunit alpha | TCA cycle |
